# Disinfection and damage of nontuberculous mycobacteria by different UV wavelengths

**DOI:** 10.1128/aem.01078-26

**Published:** 2026-07-31

**Authors:** Yijing Liu, Eunice Kum, Richard Robinson, Natalie Hull

**Affiliations:** 1 College of Engineering, Department of Civil, Environmental and Geodetic Engineering, The Ohio State University 2647, Columbus, Ohio, USA; 2 College of Arts and Sciences, Department of Microbiology, The Ohio State University 2647, Columbus, Ohio, USA; 3 Department of Microbial Infection and Immunity, The Ohio State University 2647, Columbus, Ohio, USA; 4 The Sustainability Institute, The Ohio State University 2647, Columbus, Ohio, USA; Indiana University Bloomington, Bloomington, Indiana, USA

**Keywords:** UV disinfection, far-UVC disinfection, nontuberculous mycobacteria, morphology, clinically relevant

## Abstract

Nontuberculous mycobacteria (NTM) infection is a rising global concern and a severe opportunistic waterborne infectious disease. NTM has frequently been found in municipally treated water. Distinct colony morphotypes of NTM have different cell structure properties and, therefore, different resistance to disinfectants, which has given rise to the need to further explore mechanisms that underlie mycobacterial damage by ultraviolet (UV) radiation. Several studies have investigated NTM response to 254 nm UV, but their reaction to 222 nm is largely unknown. This study investigated the treatment efficiency of different UV wavelengths (222 nm emitted by a KrCl excimer lamp and 254 nm emitted by a low-pressure Hg lamp) on inactivating non-pathogenic NTM (*Mycobacterium smegmatis*) and clinically relevant *Mycobacterium intracellulare*, and damaging *rpoB* and *hsp65* genes in smooth and rough morphotypes of these two NTM species. Results indicated that non-clinically relevant *M. smegmatis* grows faster than clinically relevant *M. intracellulare* and is less resistant to both UV wavelengths. Cell structure was found to play an important role in the resistance of NTM to UV, where rough morphotypes without glycopeptide lipids were more resistant. Gene damage analyzed by quantification of *rpoB* and *hsp65* genes indicated that 222 nm caused slightly more gene damage than 254 nm, and smooth morphotypes exhibited higher gene damage than rough morphotypes. Exploring UV mechanisms across different NTM species, morphotypes, and their genes will enhance monitoring and control of NTM in water systems, as well as targeting UV development toward specific pathogens to minimize health threats posed by NTM.

**IMPORTANCE:**

Nontuberculous mycobacteria (NTM) are found naturally in the environment and in treated water. NTM can cause opportunistic pathogen infections, which are a growing waterborne public health burden. Disinfection by ultraviolet (UV) light is a possible solution. Here, we showed that cell colony morphotype contributed to UV disinfection resistance, where rough morphotypes without glycopeptide lipids were more resistant to both disinfection and gene damage by both wavelengths we studied. Among wavelengths, 222 nm caused slightly more gene damage than 254 nm. Understanding the response of different NTM cell types and components to different wavelengths of UV light will inform the design and optimization of effective and efficient water treatment systems.

## INTRODUCTION

Nontuberculous mycobacteria (NTM) are opportunistic pathogens (OPs) that are widely distributed in the environment, particularly in water sources (1–3). The Centers for Disease Control and Prevention identified NTM as a predominant cause of hospitalizations and mortality. In 2024, NTM represented the highest cost among waterborne infectious diseases in the United States, with an estimated economic burden of $1.53 billion (4, 5). *Mycobacterium avium* complex (MAC) infections have been shown to most frequently cause pulmonary disease (6, 7). A closely related species of MAC, *Mycobacterium intracellulare*, often presents with higher severity and more prevalent lung disease (8). NTM has been isolated from diverse sources, including primarily aquatic environments, can multiply in water distribution systems when they persist after water treatments, and can increase in prevalence with distance from the treatment facility (9–11). The unique characteristics of NTM, including their waxy, hydrophobic, and lipid-rich cell wall with low permeability and their ability to form biofilms, facilitate their persistence in environments and confer resistance to disinfectants (12). The enrichment of mycolic acids in mycobacterial cell walls and membranes consists of lipids, glycolipids, and secreted proteins that promote adherence to surfaces and aggregation in liquid media (13). The glycopeptidolipids (GPLs) in some strains of NTM cell walls consist of a lipopeptide core and a long-chain fatty or mycolic acid and contribute to their pathogenicity and persistence, including antibiotic resistance and biofilm formation (10, 12, 14–16). The extracellular matrix of biofilms acts as a physical barrier to prevent the penetration of disinfectants and reduce NTM’s susceptibility (17). Both *Mycobacterium avium* and *Mycobacterium smegmatis* exhibit sliding motility, which has also been linked to their ability to form biofilms on water distribution system surfaces (14). Phthiocerol dimycocerosates (PDIMs) and acyl trehalose glycolipids located in the outer membrane of *Mycobacterium tuberculosis* act as critical virulence factors. PDIMs create an impermeable barrier, mask the bacteria, and interfere with host immune signaling pathways. PDIM, together with trehalose dimycolate, disrupts phagosome-lysosome fusion and necrotizes granuloma, facilitating bacilli release into the airways for subsequent exhalation and transmission (18). In response to disinfectants in drinking water systems, NTM have also been shown to evade and replicate inside certain free-living amoebae species (1, 3, 12, 19).

*M. smegmatis* has been used as a model to study NTM due to its non-pathogenic and fast-growing nature (20). Rapidly growing *M. smegmatis* accumulates high levels of GPLs (21). Some NTM isolates exhibit distinct or multiple colony morphologies, commonly appearing as smooth and rough morphotypes, which are related to GPL. Smooth morphotypes have a uniform and glossy appearance and have GPLs, while rough morphotypes have an irregular, dry, and corded appearance and lack GPLs. Both *M. smegmatis* and MAC exhibit multiple colony morphologies (14, 22). Research suggests that smooth *M. avium* strains exhibit increased GPL production and greater sliding motility, resulting in more pronounced biofilm formation compared to rough variants (23).

Inhalation of mycobacteria aerosols from environmental sources, such as the persistence of NTM in water supply systems, is the primary exposure route of pulmonary infection (24–27). Studies have also shown that CT scans of over 90% of patients with pulmonary NTM infection exhibit evidence of gastric content aspiration (8). A previous study found a positive association between MAC pulmonary disease and NTM isolated from shower aerosols (28). Common approaches such as filtration, monochloramine, chlorine, and ozone prove effective against various waterborne fecal pathogens (29). However, OPs, such as *Legionella pneumophila*, *Mycobacterium avium*, and *Pseudomonas aeruginosa*, are commonly found in plumbing systems and can increase in prevalence with distance from treatment plants (9, 30, 31). These organisms are associated with human infections, including pulmonary infections that may occur through inhalation of contaminated aerosols. The significance of biofilms in water distribution systems and premise plumbing is recognized for the persistent colonization and proliferation associated with OPs (3, 32), particularly mycobacteria (33). Both biofilms and certain free-living amoebae species can protect NTM from residual disinfectants in water distribution systems and building plumbing, limiting the efficacy of disinfectants like chlorine against these pathogens (34).

Ultraviolet (UV) light has the ability to penetrate the cell wall of microorganisms and be absorbed by the proteins and nucleotides constituting the cell’s components. Proteins, which comprise a large proportion of microbial cells, preferentially absorb UV at wavelengths below 230 nm (35), which can result in irreparable damage by direct absorption of UV photons by amino acids through the formation of protein-protein crosslinks and oxidation of amino acid chains (36–38), while nucleic acids primarily absorb light at a wavelength of 260 nm, which results in DNA or RNA damage that renders cells unable to reproduce but may be repaired (39). UV-C radiation at 254 nm emitted by conventional mercury lamps has demonstrated well-known efficacy in fatally harming microbial biomolecules, inducing thymine cross-linking residues in nucleotides, and disrupting the proliferation of microorganisms (40). Excimer lamps present a promising technology as a potentially safer alternative to conventional mercury lamps (41), with 222 nm emission from KrCl lamps proving comparable inactivation efficiency on bacteria and viruses (38, 42–44) and higher inactivation efficiency to adenovirus, which has a higher proportion and infection importance of proteins (36). Several studies have evaluated the effectiveness of UV irradiation for inactivating NTM species. Early work by Bohrerova and Linden investigated DNA damage and repair in *Mycobacterium terrae* following exposure to low- and medium-pressure mercury UV lamps, highlighting the role of UV-induced DNA damage in inactivation mechanisms. More recent studies have explored alternative UV sources, such as UV-LEDs, with Song et al. reporting inactivation kinetics of *Mycobacterium abscessus* using 280 nm UV-LEDs in water. Other studies have focused on clinically relevant species in drinking water systems; for example, Schiavano et al. examined the disinfection of *Mycobacterium avium* subspecies *hominissuis* using low-pressure UV lamps. In addition, Lee et al. evaluated the UV inactivation of multiple mycobacterial species, including *M. avium*, *Mycobacterium fortuitum*, *M. intracellulare*, and *Mycobacterium lentiflavum*, under both low- and medium-pressure UV irradiation (24–27).

Despite their clinical significance and ubiquity, existing studies on UV inactivation of NTM remain limited in scope. Prior work has primarily focused on conventional low- and medium-pressure UV systems, with no attention to emerging wavelengths such as 222 nm. In addition, comparative evaluations across different colony morphotypes and between clinically relevant and non-pathogenic NTM species are lacking. This work evaluated the effects of two UV wavelengths (222 nm and 254 nm) on the inactivation of two NTM species, nonpathogenic *M. smegmatis* and the clinically relevant *M. intracellulare*, and examined damage to the *rpoB* and *hsp65* genes in both smooth and rough morphotypes. To provide a visual overview of the study design and experimental workflow, the graphical abstract has been included in the supplemental material. This study addressed the research gap by investigating the effects of different UV wavelengths on NTM at cellular and biomolecular levels and explored variations among colony morphotypes, strains, and clinical relevance of the species. By understanding the properties of NTM and their interaction with UV wavelengths, potential implications for public health and environmental management strategies can be applied.

## MATERIALS AND METHODS

### NTM strains and *M. intracellulare* morphotypes

In this study, we utilized two strains of non-pathogenic BSL-1 *Mycobacterium smegmatis* displaying a smooth morphotype, namely *M. smegmatis* 19420 (ATCC) and *M. smegmatis* 14468 (ATCC), and three colony morphotypes (parent, smooth, and rough) of pathogenic clinical isolates of *Mycobacterium avium-intracellulare* (*M. intracellulare*). *M. intracellulare* isolates were obtained as previously described (45) under approved protocols from children who had an abscess removed (parent strain, consisting of a mixture of rough and smooth colony morphotypes) and subsequently selectively cultured to generate smooth and rough colony morphotypes.

### Growth medium preparation and bacterial cultivation

Glycerol stocks of *M. smegmatis* strains were prepared according to ATCC protocols and were stored at −80°C. *M. smegmatis* and *M. intracellulare* were grown on Difco Middlebrook 7H9 broth and 7H10 agar. To make 7H9 broth, 4.7 g 7H9 agar powder and 900 mL deionized water were combined in a glass bottle and autoclaved on a liquid cycle at 121°C for 10 min. After cooling to around 50°C–55°C, 100 mL of filtered OADC (oleic acid-albumin-dextrose-catalase) enrichment was aseptically added mixed with 2.5 mL of Tween 80 to prevent cell aggregation. For 7H10 agar, 5 mL glycerol was added aseptically into 19 g of 7H10 agar powder and 900 mL deionized water in a glass bottle and boiled for 60 s before being autoclaved at 121°C for 10 min. A total of 100 mL of filtered OADC enrichment was added aseptically when cooled to around 50°C–55°C. A total of 12 mL of 7H10 agar broth was added to 100 mm × 15 mm plates. Broth and agar plates were stored at 2°C–8°C until use for culture or enumeration of colony-forming units (CFU), or used fresh for the rough morphotype of *M. intracellulare*. A 0.01 mL of bacterial glycerol stocks was added to a 6 mL aliquot of 7H9 broth to an individual Erlenmeyer flask and incubated at 37°C in a shaking incubator. For growth characterization, bacterial cultures were incubated at 37°C in a shaking incubator. Flasks were wrapped in aluminum foil to maintain dark conditions and prevent photoreactivation. Growth was monitored over approximately one week using optical density (OD) measurements. In parallel, samples were periodically collected and quantified using spot plating to establish growth curves. Detailed sampling time points and corresponding data are provided in the Supplemental Information (Tables S3 to S9).

### Optical density

OD was measured using the Thermo Scientific NanoDrop 2000/2000c Spectrophotometer at each time point throughout the experiment. A total of 20 mL of samples from each bacterial flask were transferred aseptically to PCR tubes, and 2 mL of each sample were used to measure OD at 600 nm at each timepoint, as detailed in Tables S3 to S7. All studies used 7H9 growth liquid medium as the negative control to confirm sterility, and OD_600_ for the negative control was subtracted from the samples. Figure S1 illustrates the temporal variations of optical density over time.

### Microorganism enumeration

CFU were measured concurrently with OD_600_ throughout growth curve experiments. Sterile normal saline solution was used to prepare serial dilutions. A total of 10 mL of each dilution was spotted on 7H10 agar plates, with 10 replicate spots for each dilution (46). The plates were then incubated at 37°C, followed by the quantification of CFUs after approximately 35 h for *M. smegmatis* and 175 h for *M. intracellulare*. The averaged CFU values for each replicate of *M. smegmatis* and *M. intracellulare* are summarized in Tables S8 and S9. Figure S2 illustrates the temporal variations of cell counts over time.

### UV dose determination

**Fig 1:**
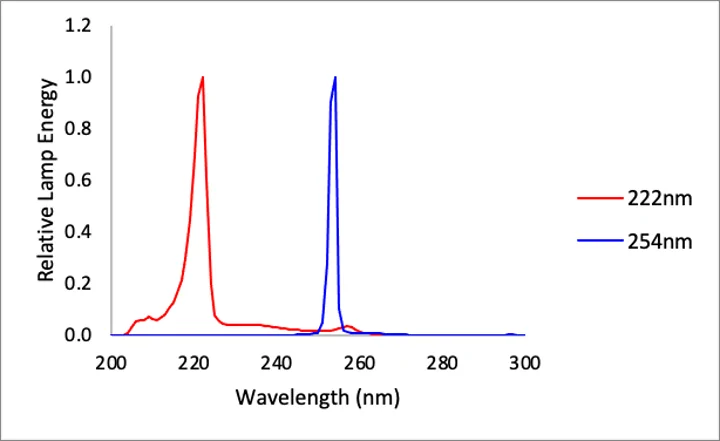
Emission spectra from low-pressure (LP) mercury lamp (254 nm) and krypton-chloride excimer (KrCl*) lamps (222 nm).

Standardized polychromatic UV dose calculations described by Linden and Darby (47), Bolton and Linden (48), and available in an open-source protocol (49) were used to calculate sample exposure times. After pre-warming lamps, total incident irradiance was measured using an International Light ILT-5000 radiometer set to the appropriate factor for peak emission wavelength with a SED240W photodetector and corrected for several factors, including the Petri Factor to compensate for non-uniformity across the sample surface, correcting for beam divergence according to experimental geometry, accounting for polychromatic sample absorbance (Agilent Cary 4000 UV-Vis Spectrophotometer), accounting for reflection factor at peak wavelength, and accounting for polychromatic lamp emission measured by Ocean Optics HDX UV-VIS Spectroradiometer (Fig. 1). These adjustments were used to calculate exposure times necessary to achieve specific pre-determined UV doses.

### UV exposures

*M. smegmatis* was grown for approximately 35 h, and *M. intracellulare* was cultured for 175 h until reaching the exponential phase before using them for UV exposure. Exponential phase cells were exposed to UV fluences ranging from 0 to 120 mJ/cm^2^ with a target starting concentration at around 10^7^ CFU/mL. Cultures were individually transferred into 50 mL centrifuge tubes and centrifuged at 5,000 rpm for 5 min. After discarding the supernatant and washing the pellets with 10 mL of PBS by vortexing, the tubes were centrifuged for an additional 5 min. Subsequently, 50 mL of PBS was added to the centrifuge tubes containing the pellet, followed by vortexing. Working solutions were prepared by adding 10 mL of the resuspended samples into 100 mL of PBS. A total of 5 mL of the working solution was added to each 35 × 10 mm Petri dish, which was placed on a stir plate with quiescent stirring for the calculated time to achieve a given UV dose for a given lamp. Unexposed samples were used as the 0 mJ/cm^2^ dose condition. UV experiments were conducted fully in the dark environment, and samples were covered by aluminum foil to avoid photo repair before transferring samples or plating. Aliquots of samples with and without UV exposure were serially diluted, and 10 μL samples were immediately plated on 7H10 agar plates, and aliquots of samples were transferred into labeled centrifuge tubes, covered with aluminum foil to prevent photo repair, and stored at −20°C until DNA extraction. For culture-based enumeration, the detection limit was determined based on a minimum detectable count of 1 colony, corresponding to 100 CFU/mL (49).

### DNA extraction and qualitative PCR

DNA was extracted using the extraction protocol previously outlined (50), modified to use phenol:chloroform:isoamyl alcohol. Qualitative PCR (qPCR) was employed to assess damage to target genes, including RNA polymerase β subunit (*rpoB*) (723 bp) and heat shock protein 65 (*hsp65*) (441 bp), by examining their reduced amplification compared to samples without UV exposure. Qubit Fluorometer with 1× dsDNA high sensitivity assay kits was used to quantify the DNA concentration at each UV dose for 254 nm and 222 nm wavelengths. DNA concentration change was summarized in Fig. S7. All qPCR assays were conducted in triplicate using the QuantStudio 6 Flex Real-Time PCR System (Thermo Fisher Scientific, USA). Amplicon length and primer information for both genes are summarized in Table S10. Reaction preparation protocol for *rpoB* gene and *hsp65* gene followed a previous study (51, 52). The qPCR cycling protocols are listed in Tables S11 and S12. Standard curves (gBlocks, IDT) were designed and used to determine the copy numbers of each target *rpoB* gene and *hsp65* gene (Text S1) and to determine amplification efficiency using the slope of the standard curves fitted to a linear model, equal to 10^(−slope)^ − 1. The efficiencies for *rpoB* and *hsp65* were 0.93 and 1.16, which are in the typical amplification efficiency range. Log inactivation was calculated as log_10_ (*N*_0_/*N*), where *N*_0_ and *N* represent gene copy numbers before and after UV exposure, respectively, as determined by qPCR. Rapid sequencing DNA V14- barcoding (SQK-RBK114.96) was used for library preparation and loaded to a flowcell in MK1C sequencer for sequencing, following the protocols provided on the Nanopore website. The wf-amplicon workflow (https://labs.epi2me.io/workflows/wf-amplicon/) was used to analyze the reads created after sequencing with default settings for parameters including minimum read length (300) and minimum coverage (20). Amplicon length under each dose with 254 nm and 222 nm for each species was summarized in Fig. S8 and S9.

### Dose response modeling

Growth and disinfection (inactivation and gene damage) data were fit to a log-logistic model (Equation 1), setting *c* as 0 in R using the “drm” function from the “drc” package (R Version 3.0-1) (53). LI is Log10 inactivation for cell inactivation or Log10 gene damage for qPCR quantification; *b* is the slope of the curve at the middle point (*e*); *c* is the minimum value that could be obtained at 0 dose, which we set to 0, resulting in a three-parameter rather than a four-parameter model; and *d* is the maximum value. Parameters of *b*, *d*, and *e* with their standard errors obtained from regression models are summarized in Tables S1, S13 and S14. Statistical significance of differences was assessed through the lack of overlap between the range of standard errors of model parameters.


(1) $$LI=c+\frac{d-c}{1+\exp(b\times (\log\text{fluence}-\log e))}$$

Based on the fitted model parameters, *D*_1_ and *D*_2_ were calculated as the UV fluences required to achieve 1-log₁₀ (90%) and 2-log₁₀ (99%) reductions in inactivation or gene damage, respectively. The corresponding inactivation rate constants (*k*_1_ and *k*_2_) were defined as the reciprocals of *D*_1_ and *D*_2_.

### Electrical efficiency

The formula for the electrical energy per order (EEO, kWh/m^3^/order) calculation is


(2) $$\text{EEO}=\frac{A\times D_{N}}{3.6\times 10^{6}\times V\times C\times WF}$$

as previously calculated by reference 54. Here, *A* represents the irradiated surface area in square centimeter calculated by the diameter of the Petri dish (9.62 cm^2^). V is the sample volume in liters (0.003 L). *D*_*N*_ is the UV dose (mJ/cm^2^) required for obtaining a specific log reduction, which is the same as *D*_1_ or *D*_2_ for 1 or 2 log, respectively, of cells or gene damage, as summarized in Tables S13 and S14. *C* is the wall plug efficiency given by the manufacturer (0.035 for 254 nm, and 0.1 for 222 nm [54–61]). WF is the water factor, accounting for the UV absorbance and depth of the water, which we averaged across three biological duplicate UV experiments. The factor 3.6 × 10^6^ converts between hours and seconds, milliwatt and kilowatt, and liter and cubic meter.

## RESULTS

### Inactivation of non-pathogenic *Mycobacterium smegmatis* strains and clinically relevant *Mycobacterium avium-intracellulare* morphotypes

**Fig 2:**
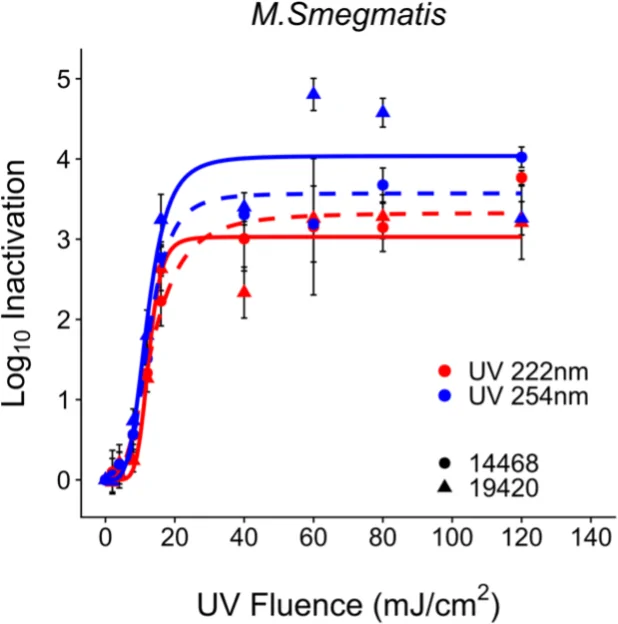
UV dose response (222 nm, red vs 254 nm, blue) with log-logistic model for log10 reduction of *M. smegmatis* 19420 and 14468. Error bars represent the standard error of the mean (SEM) of three biological replicates at each UV dose. The solid line represents *M. smegmatis* 19420, while the dotted dashed line represents *M. smegmatis* 14468.

**Fig 3:**
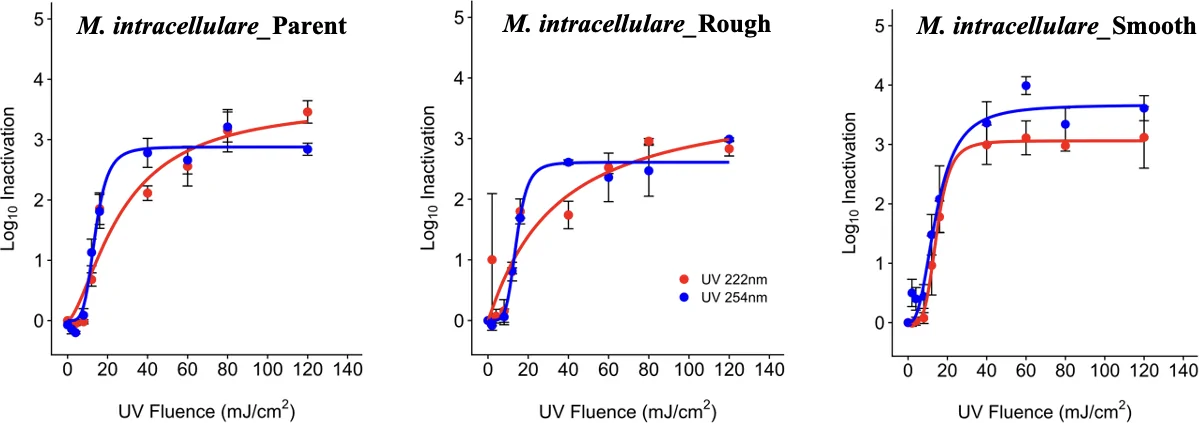
UV dose response (222 nm, red vs 254 nm, blue) with log-logistic model for log10 reduction of morphotypes of *M. intracellulare* (*M. intracellulare*_Parent [left], *M. intracellulare*_Rough [middle], *M. intracellulare*_Smooth [right]). Error bars represent SEM of three biological replicates at each UV dose.

**Fig 4:**
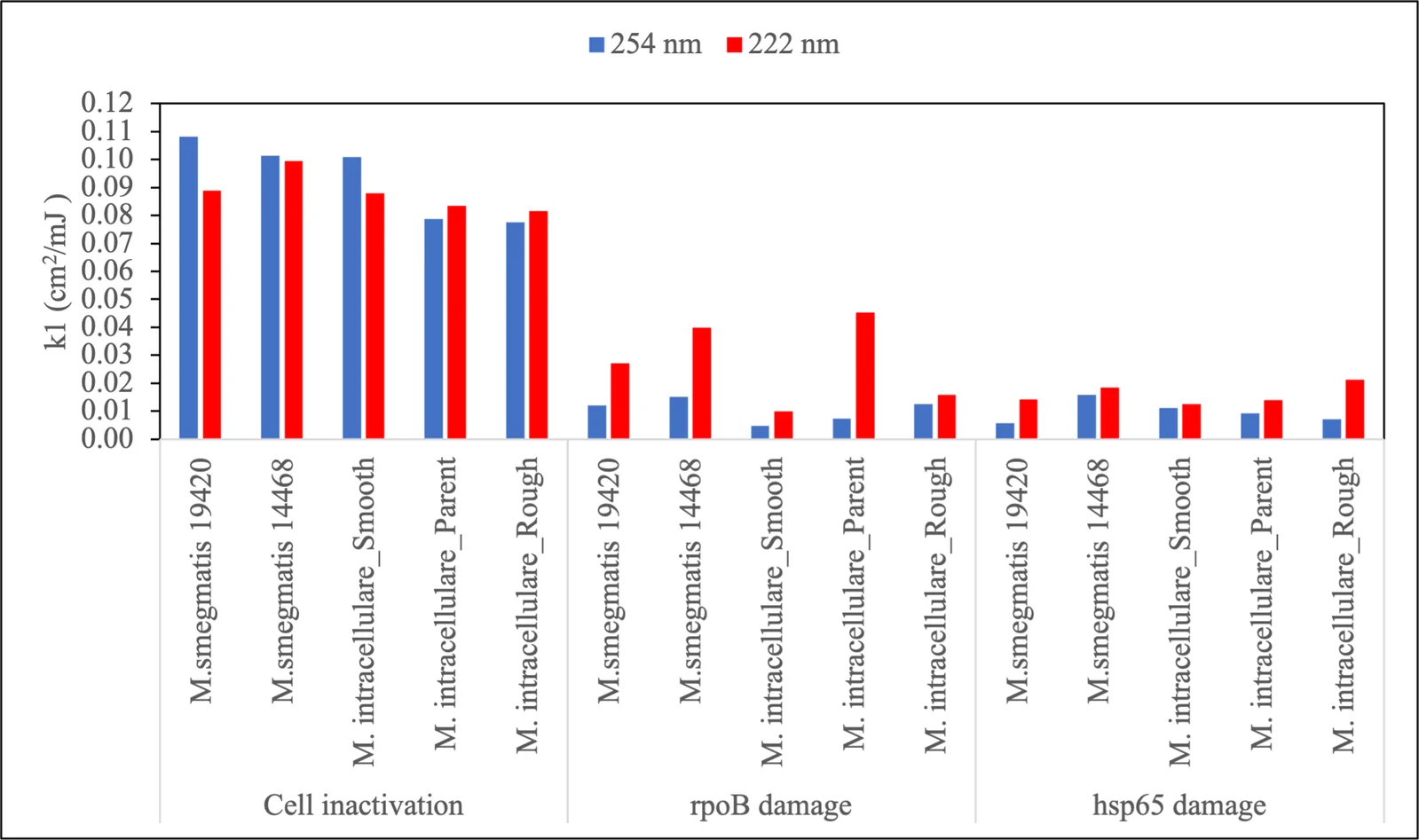
*k*_1_ for 1-log10 reduction of *M. smegmatis* and *M. intracellulare* inactivation, *rpoB* and *hsp65* gene damage with 254 nm (blue) and 222 nm (red) UV.

To explore the mechanisms that underlie differences in UV disinfection resistance between NTM species, strains, and colony morphotypes, we conducted collimated beam UV experiments with exponential phase cells under 254 nm or 222 nm within the range of 0 to 120 mJ/cm^2^. Figure 2 shows the UV dose curves for *M. smegmatis*, while Fig. 3 shows the UV dose curves for *M. intracellulare*. Parameters of fitted log-logistic models with their standard errors are summarized in Table S13 for *M. smegmatis* and *M. intracellulare*. Rate constant *k*_1_ for 1-log reduction was plotted in Fig. 4.

For nonpathogenic *M. smegmatis* strains, which have smooth morphotypes and contain GPL, the UV dose responses did not statistically significantly differ between the two strains. For *M. smegmatis* 19420, 9.24 mJ/cm^2^ of 254 nm (*k*_1_ = 0.11 cm^2^/mJ) and 11.26 mJ/cm^2^ (*k*_1_ = 0.09 cm^2^/mJ) of 222 nm were needed for 1-log reduction (*D*_1_). For *M. smegmatis* 14468, 9.88 mJ/cm^2^ of 254 nm (*k*_1_ = 0.10 cm^2^/mJ) and 10.07 mJ/cm^2^ (*k*_1_ = 0.10 cm^2^/mJ) of 222 nm were needed for 1-log reduction inactivation. *D*_2_ even had a smaller difference among the strains and wavelengths (Table S13). The maximum log inactivation due to tailing effects was higher for 254 nm (4.04 and 3.57) than 222 nm (3.03 and 3.33) for both *M. smegmatis,* indicated by parameter *d* in Table S13.

For *M. intracellulare*, the smooth morphotype (9.91 mJ/cm^2^) was more significantly sensitive to 254 nm UV than both parent (12.72 mJ/cm^2^) and rough (12.89 mJ/cm^2^) morphotypes, which were similar to each other, and smooth had similar UV sensitivity to both strains *M. smegmatis*, based on *D*_1_ and *k*_1_ (Table S13 and Fig. 4). When comparing *k*_2_ (Tables S13 and S14), however, differences were not significant due to the nonlinearity of dose responses, indicating the importance of checking multiple kinetic parameters to capture all the variations. Sensitivity to 222 nm UV was similar among the three morphotypes, as they had similar *D*_1_ and *k*_1_ (Table S13 and Fig. 4). However, rough (36.94 mJ/cm^2^) required a significantly higher fluence to obtain a 2-log reduction (*D*_2_) compared to parent (17.15 mJ/cm^2^) and smooth (15.95 mJ/cm^2^) morphotypes.

Comparing *D*_1_ (Table S13) among *M. smegmatis* or *M. intracellulare* between wavelengths, *D*_1_ was lower at 254 nm for *M. smegmatis* than at 222 nm, indicating the higher sensitivity of *M. smegmatis* to 254 nm. *D*_1_ was similar for all MAC strains between 254 nm and 222 nm, except *M. intracellulare*_Smooth at 254 nm, with a lower value. Comparing *k*_1_ between *M. smegmatis* and *M. intracellulare*, the sensitivity of both strains of *M. smegmatis* was similar to smooth *M. intracellulare*, and together these smooth NTM morphotypes were more sensitive than both rough and parent morphotypes. These findings suggest that, within the two NTM species examined in this study, differences between species did not result in substantial variation in UV sensitivity, whereas cell properties related to GPL contents and resulting colony morphotype appeared to play a more influential role. Tailing effects shown in dose responses and modeled by *d* in Table S13 showed that smooth at 254 nm had a higher maximum log reduction of 3.67 compared to 222 nm (2.99). While rough indicated a higher maximum log reduction of 3.86 by 222 nm over 254 nm (2.61).

### DNA damage of non-pathogenic *Mycobacterium smegmatis* strains and clinically relevant *M. intracellulare* morphotypes

qPCR was employed to assess damage to target genes with *rpoB* and *hsp65* by examining their reduced amplification. Standard curves with serially diluted extracted DNA from NTM stocks and gblock standards are shown in Fig. S3 and S4 for *rpoB* and *hsp65*, respectively. Parameters of fitted log logistic models with their standard errors are summarized in Table S14 for *M. smegmatis* and *M. intracellulare*. Gene damage for *rpoB* and *hsp65* for *M. smegmatis* and *M. intracellulare* is shown in Fig. S5 and S6. Both *rpoB* and *hsp65* DNA damage of *M. smegmatis* and *M. intracellulare* increased with increasing doses for both wavelengths. Rate constant *k*_1_ for 1-log reduction was plotted in Fig. 4.

For nonpathogenic *M. smegmatis*, gene damage by 222 nm and 254 nm was initially rapid up to 20 mJ/cm^2^. Comparing *D*_1_ for both *rpoB* and *hsp65*, UV fluence required by 222 nm was much lower for both strains of *M. smegmatis* indicating the more effective impacts on *rpoB* and *hsp65* by 222 nm over 254 nm, although 2-log reduction needs higher UV doses to achieve or is hard to achieve for either wavelength, indicated by *d* and *e* in Table S14. Comparing rate constant kinetics *k*_1_ among *rpoB* and *hsp65* (Fig. 4), in *M. smegmatis* 19420, 254 nm had a lower rate constant with higher doses required for both *rpoB* and *hsp65* damage, while in *M. smegmatis* 14468, *hsp65* was damaged similarly by 222 nm and 254 nm, but *rpoB* was more resistant to 254 nm.

Among *M. intracellulare* morphotypes, due to the irregularity of the *rpoB* gene damage log logistic dose response for *M. intracellulare* parent at 254 nm, we also used linear regression to estimate the rate constant as 0.0072 cm^2^/mJ. Comparing *k*_1_ for *rpoB* between 254 nm and 222 nm for *M. intracellulare* (Fig. 4), *k*_1_ by 222 nm (0.01 cm^2^/mJ) was higher than 254 nm (0.005 cm^2^/mJ) for smooth, *k*_1_ by 222 nm (0.05 cm^2^/mJ) was higher than 254 nm (0.007 cm^2^/mJ) for parent while rough had similar kinetics for 222 nm (0.016 cm^2^/mJ) and 254 nm (0.013 cm^2^/mJ). For *hsp65*, the rate constant was similar for smooth and parent around 0.01 mJ/cm^2^ for both 254 nm and 222 nm, while rough had a higher rate constant *k*_1_ (0.021 cm^2^/mJ) at 222 nm than at 254 nm (0.007 cm^2^/mJ). The parent also had a higher rate constant *k*_1_ (0.014 cm^2^/mJ) at 222 nm than at 254 nm (0.009 cm^2^/mJ).

Comparing *M. smegmatis* with *M. intracellulare* morphotypes, for *hsp65* treated by 254 nm, *k*_1_ of *M. intracellulare* parent or smooth (~0.01 cm^2^/mJ) was higher than *M. smegmatis* 19420 (0.006 cm^2^/mJ) and similar to *M. smegmatis* 14468 (0.013 cm^2^/mJ), and *k*_1_ of *M. intracellulare* rough (0.007 cm^2^/mJ) was similar to *M. smegmatis* 19420 (0.006 cm^2^/mJ). *k*_1_ at 222 nm was similar for *M. smegmatis* 19420 and *M. intracellulare* parent or smooth (~0.014 cm^2^/mJ), and *k*_1_ at 222 nm for *M. intracellulare* rough (0.021 cm^2^/mJ) was similar to *M. smegmatis* 14468 (0.018 cm^2^/mJ). For *rpoB* treated by 254 nm, *k*_1_ of *M. intracellulare* rough (~0.013 cm^2^/mJ) was similar to both *M. smegmatis* 19420 and *M. smegmatis* 14468. For *k*_1_ of *M. intracellulare* parent (0.007 cm^2^/mJ) or smooth (0.005 cm^2^/mJ), they were lower than *M. intracellulare* rough and both *M. smegmatis*. For *rpoB* treated by 222 nm, *k*_1_ of *M. intracellulare* parent (0.045 cm^2^/mJ), *M. smegmatis* 19420 (0.027 cm^2^/mJ), and *M. smegmatis* 14468 (0.040 cm^2^/mJ) were higher than *M. intracellulare* smooth (0.010 cm^2^/mJ) and rough (0.016 cm^2^/mJ; Table S14 and Fig. 4). Again, these findings indicated the minor effect on UV dose damage by strain. Rate constants *k*_1_ for *hsp65* at 222 nm were lower than *rpoB* for both *M. smegmatis* and *M. intracellulare* morphotypes, indicating higher UV sensitivity of *rpoB*. One important observation was that DNA concentration decreased following UV exposure (Fig. S7), suggesting increasing UV-associated DNA damage or fragmentation, which should be considered when understanding UV effects. However, no substantial change in amplicon read length distribution was observed after UV exposure (Fig. S8 and S9). Further investigation is needed to better understand the mechanisms underlying DNA damage and to determine whether cellular characteristics or other factors may influence these observations.

### Electrical energy comparisons

**Fig 5:**
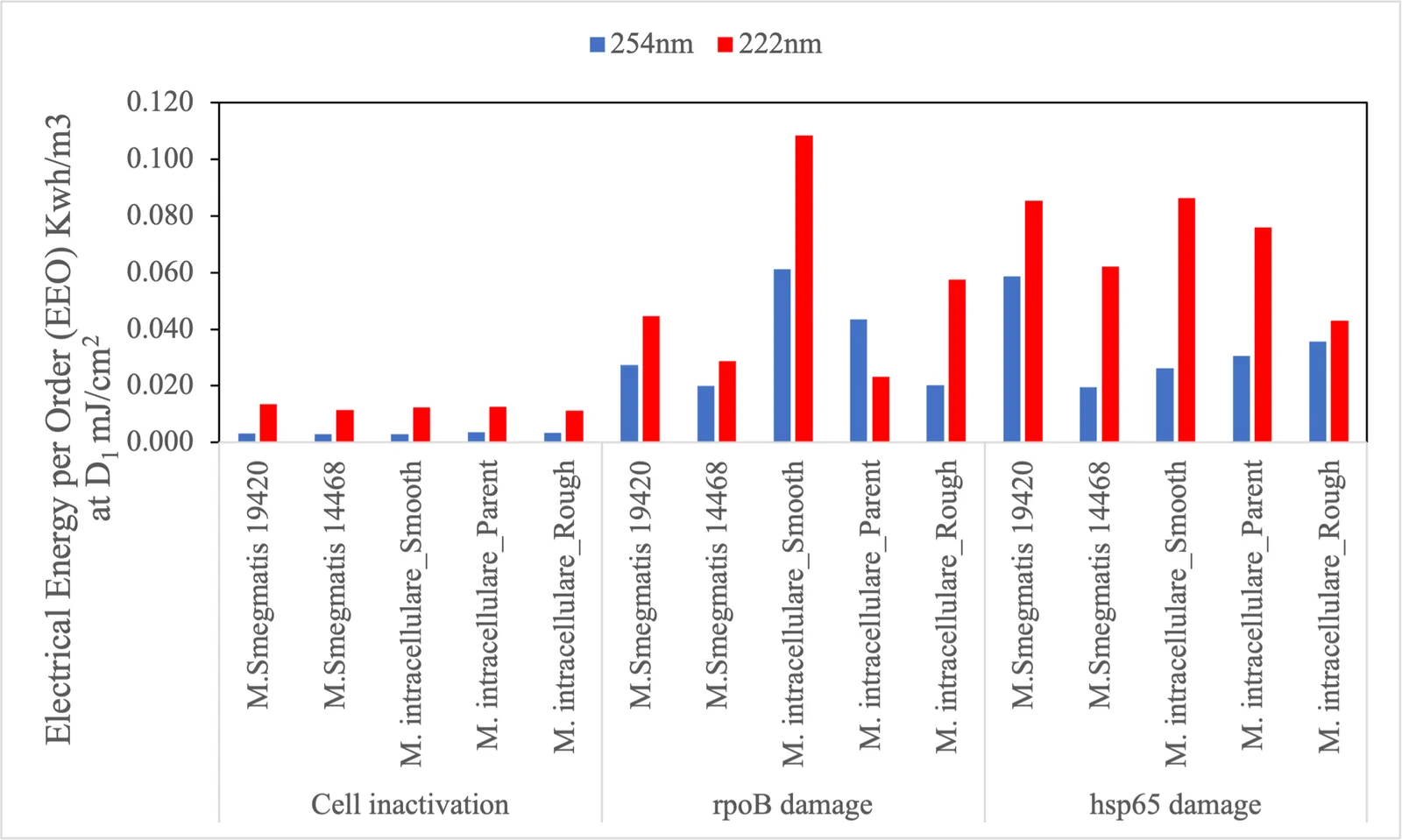
Electrical energy per order at D1 of *M. smegmatis* and *M. intracellulare* inactivation, *rpoB* and *hsp65* gene damage with 254 nm (blue) and 222 nm (red) UV.

EEO at 1-log reduction for NTM cell inactivation and *rpoB* or *hsp65* gene damage is summarized in Fig. 5, and the energy consumption for all conditions was between 0.003 and 0.108 kWh/m^3^. NTM inactivation took lower energy consumption compared to gene damage for all NTM strains. Energy consumption was similar for *rpoB* and *hsp65* gene. Energy efficiency for 222 nm was lower than 254 nm within the variations of EEO at 254 nm overlapped with EEO at 222 nm.

## DISCUSSION

### Sensitivity of different NTM to different UV wavelengths

Understanding the mechanisms underlying NTM response to UV wavelengths is crucial for various applications, including disinfection processes and environmental control. Several studies utilizing 254 nm have been conducted with *Mycobacterium spp*. For example, secondary wastewater effluent spiked with *M. terrae* (smooth morphotype) was exposed to 254 nm low-pressure (LP) UV ranging from 5 to 60 mJ/cm² (62), where the rate constant was 0.16 cm²/mJ, which was slightly higher than the rate constant for our study (around 0.1 cm²/mJ for both *M. smegmatis* and *M. intracellulare*). Tailing also slightly differed and was observed after 20 mJ/cm² in the previous study and around 13 mJ/cm^2^ in our study (Table S13). Tailing is commonly observed in mycobacteria disinfection dose responses and may be attributed to aggregation due to the unique cell walls containing fatty mycolic acid. In another study where tap water was spiked with 14 strains of *M. avium* subspecies *hominissuis* exhibiting smooth colonies and exposed to submerged 254 nm UV doses of 0–192 mJ/cm^2^, susceptibility varied among strains, while in our study, non-pathogenic *M. smegmatis* and *M. intracellulare*_Smooth were similar to each other and more sensitive to UV than *M. intracellulare*_Parent and *M. intracellulare*_Rough. For five strains, 3-log reduction occurred at ≤32 mJ/cm^2^, and 13 of the 14 strains were disinfected below detection from the initial concentration of 10^5^ CFU/mL. In our study, 3-log reduction occurred at around 16 mJ/cm^2^, but no strain or morphotype was disinfected below detection from the initial concentration of 10^7^ CFU/mL due to tailing. Instead of doing a collimated beam study as we did to ensure highly standardized and repeatable results (48), the previous study immersed UV lamps onto cylindrical glass tubes in which the UV light disperses in all directions and provides broad exposure to water. The differences in reactor configuration and lower initial concentration in their study could explain the lack of tailing in their study. In another study, water spiked with clinical isolates of *M. abscessus*, *M. avium*, and *Mycobacterium chimaera* indicated that two different point-of-use 254 nm UV systems achieved an average NTM log reduction of 0.89 and 1.86 with an exposure time of 90 s, by the suspended and submerged systems, respectively (40). In our study, a comparable exposure time corresponded to an irradiance of 8–16 mJ/cm² for 254 nm and 4–8 mJ/cm² for 222 nm. For cell inactivation, these approximate exposure times caused log reductions ranging from 1 to 3 under 254 nm and 0.5–1 under 222 nm for *M. smegmatis*, and 1–2 under 254 nm and around 0.2–0.6 under 222 nm for *M. intracellulare*.

Earlier studies also explored UV disinfection of *Mycobacterium spp*. in water by LP and multi-wavelength medium-pressure (MP) UV irradiation (63, 64). Zuzana et al. treated *M. terrae* with LP and MP and found linear rate constants of 0.16 cm²/mJ and 0.17 cm²/mJ, respectively, which did not significantly differ despite wavelength differences. These rate constants are within the same order of magnitude of our rate constants (at 1-log reduction, we observed an average value of 0.1 cm²/mJ across both *M. smegmatis* and *M. intracellulare* calculated by *D*_1_), but *M. terrae* was more sensitive than *M. smegmatis* and *M. intracellulare* in our study, as 10 mJ/cm² resulted in a 2-log reduction by both UV lamps. In another study by Shin et al. with LP disinfection, no significant differences were observed between two morphotypes (smooth transparent and smooth opaque) of clinical isolates of *M. avium* subsp. *hominissuis* HMC02 with an initial concentration of 10^6^ CFU/mL, where a 4-log reduction required approximately 24 mJ/cm² for WT and 23 mJ/cm² for WO. A 6 mJ/cm^2^ was used for 1-log reduction with a corresponding *k*_1_ around 0.17 cm²/mJ. In our study, 4-log inactivation was not achieved due to tailing effects, likely due to our higher initial concentration. The rate constants across these studies were similar, which indicated the relatively consistent resistance of NTM to UV.

Recent studies have examined the use of light-emitting diodes (LED) for disinfecting NTM. One study utilized 280 nm UV-LED to disinfect pathogenic *M. abscessus* (Rough) in water, achieving a 2-log reduction at 10 mJ/cm², whereas here, approximately 16–20 mJ/cm² were needed to obtain a 2-log reduction for *M. smegmatis* and *M. intracellulare* with 254 nm and 222 nm. Both shouldering and tailing appeared in their UV LED dose responses, whereas we only observed tailing and no significant shouldering with LP and KrCl lamp exposures. Their inactivation rate constants of 0.36 and 0.37 cm²/mJ, determined by linear regression and Geeraerd modeling (65), were higher than the rate constant in our study for both 254 nm and 222 nm modeled with log-logistic regression. This suggests a decreased resistance of *M. smegmatis* and *M. intracellulare* to 254 nm and 222 nm UV radiation compared to *M. abscessus* to 280 nm UV radiation. Additionally, Ma et al. used 222 nm to inactivate some common pathogens and surrogates, including *M. smegmatis* 14468 that we tested here (66). The rate constant for *M. smegmatis* 14468 was around 0.015 (±0.006) cm^2^/mJ for 1-log reduction after fitting to a second-order polynomial regression model, which overlaps with its value in our study (*k*_1_ = 0.099 cm^2^/mJ).

Previous investigations for inactivating bacteria and viruses also indicated comparative energy efficiency between 222 nm KrCl excimer lamps and 254 nm LP UV lamps; they also had higher energy efficiency compared to UV LED (54, 55). EEO was also calculated (Tables S13 and S14), and the values were similar between 254 nm and 222 nm, while *M. intracellulare* _Rough, *M. intracellulare*_Smooth, and *M. smegmatis* 19420 at 222 nm required slightly higher energy.

### Structural properties and gene damage

In the NTM inactivation study, we demonstrated that smooth morphotypes containing GPL, including both *M. smegmatis* and *M. intracellulare*, are more sensitive to UV light than morphotypes lacking GPL, and 254 nm was more effective than 222 nm for rough NTM. A previous study indicated that the rough morphotype of *M. intracellulare* was associated with higher virulence and pathogenicity compared to the smooth morphotype (15), which aligns with our observation of higher resistance of *M. intracellulare* _Rough and higher resistance of pathogenic *M. intracellulare*.

So far, no study has explored the UV interactions with GPLs. We observed that the different UV sensitivity between smooth and rough *M. intracellulare* was more significant than between *M. smegmatis* and *M. intracellulare* species, which may indicate the association of disinfection resistance with the differences in cell structure (with or without GPLs). However, the underlying mechanisms remain unclear. While differences in GPL composition may contribute to this observation, other structural or physiological factors may also play a role. Different morphotypes also impact virulence, where rough morphotypes of *M. abscessus* are more infectious and persistent (15). Different morphotypes have been associated with variations in cell surface structure and physiological behavior. In this study, rough morphotypes exhibited higher UV resistance compared to smooth morphotypes, which may be related to differences in cell envelope composition. In addition, the relatively high GC content of NTM species (e.g., 69% in *M. avium* and 67% in *M. smegmatis*) may contribute to overall resistance to UV disinfection due to increased DNA stability and nucleotide photoreactivity. Additionally, high GC content of 69% in *M. avium* (67) and 67% in *M. smegmatis* (68) may contribute to overall resistance to UV disinfection due to increased DNA stability and nucleotide photoreactivity with the presence of more stable triple hydrogen bonds connecting G-C and more photoreactive TC and TT sites (69, 70). Covered by cellular components with high GC content, less UV irradiation will penetrate and reach the genome for DNA damage, which contributed to the lower observed dose response in our study. Further explorations between UV and NTM cell structures could be done to complete our understanding of UV disinfection on NTM at the molecular levels.

Compared with cell inactivation, the rate constant of NTM damage was much lower, indicating the importance of understanding the molecular interactions with UV. According to the authors’ knowledge, no study so far has explored UV effects on *rpoB* and *hsp65* gene damage or quantified the UV gene damage and indicated higher UV sensitivity of *rpoB* over *hsp65*, although previous studies have focused on the improved ability of these genes over 16S rRNA in sequencing to distinguish mycobacterial species in biofilms and water distribution systems (71–73). The housekeeping *rpoB* gene, which is responsible for coding the RNA polymerase subunit, evolved more rapidly than ribosomal genes (74). The *hsp65* gene encodes the 65 kDa heat shock protein and is responsible for protein folding and stress response (75). Previous studies mainly focused on the role of *rpoB* or hsp65 as phylogenetic markers to identify mycobacterial species, while no study so far has explored the role of *rpoB* or hsp65 in resistance to UV and the properties related to resistance, such as function, coding location, amplicon length, etc. In this study, *rpoB* and *hsp65* were selected as representative gene targets due to their conserved presence and widespread use in mycobacterial identification, enabling assessment of UV-induced gene damage at the molecular level. This study provided a first glance at the difference in UV sensitivity between them and indicated higher resistance by *hsp65*, which more in-depth research could be done in the future to deepen our understanding of NTM and their resilience to UV exposure (76–78). A comprehensive understanding of the sensitivity of these and other genes to different UV wavelengths in NTM, alongside understanding how other unique cell properties of NTM interact with UV wavelengths, will help limit the distribution of NTM in water. This knowledge will prompt the development of NTM pathogen-specific disinfection protocols, optimizing water treatment for specific microorganisms.

### Implications for water treatment

A commonly required UV dose in the United States is 22 mJ/cm^2^ in drinking water to ensure 4-log inactivation of waterborne protozoa (79), while 40 mJ/cm^2^ is widely regarded to provide 4-log inactivation for most waterborne enteric pathogens (80), but higher doses are required for more resistant microorganisms (81). Previous studies regarding pathogen regulation with UV mainly focus on 254 nm and fecal pathogens, where *Escherichia coli* was the main indicator (82); however, our results and previous literature indicate lower inactivation efficiency, especially tailing, for NTM, which can be OP that may pose a great threat to human health. NTM infection is not a nationally notifiable disease (83), and NTM monitoring and reporting for drinking water is not required in the U.S. or other countries, making it difficult to fully understand the magnitude of its prevalence and quantify risks to human health due to NTM. The high resistance and prevalence of NTM in environments or in engineered water systems, as well as the lack of surveillance of NTM prevalence and health outcomes, indicate the need for greater study to comprehensively understand these OPs to develop and apply UV technology to control NTM and minimize risks to human health.

In summary, this study provides several novel insights into UV inactivation of NTM that extend beyond prior work focused primarily on conventional UV systems. By systematically comparing two UV wavelengths (222 nm and 254 nm) across both a non-pathogenic model organism (*M. smegmatis*) and a clinically relevant species (*M. intracellulare*), we demonstrate that species-specific differences play a critical role in UV susceptibility, with *M. intracellulare* exhibiting greater resistance. Furthermore, our results highlight the importance of cell structure, showing that rough morphotypes lacking glycopeptidolipids exhibit increased resistance to UV exposure.

In addition, gene damage analysis revealed wavelength- and morphotype-dependent responses, with 222 nm inducing slightly greater damage to target genes (*rpoB* and *hsp65*) compared to 254 nm, and smooth morphotypes showing higher levels of gene damage than rough morphotypes. Together, these findings provide new evidence that both cellular structure and physiological characteristics influence UV disinfection outcomes and underscore the need to consider species and morphotype variability when evaluating emerging UV technologies for controlling NTM in water systems. It should be noted that this study was conducted in PBS as a simplified and controlled matrix to isolate the effects of UV exposure; however, it does not fully capture the complexity of real drinking water systems, where factors such as natural organic matter, particles, and biofilm formation may influence disinfection efficiency. Therefore, the results presented here may not fully represent *in situ* conditions and should be interpreted with consideration of these limitations. Future studies incorporating more representative water matrices are needed to further evaluate the applicability of these findings.

## Data Availability

The raw Nanopore amplicon sequencing reads generated in this study have been deposited in the NCBI Sequence Read Archive under BioProject accession number PRJNA1491417 and SRA accession numbers SRR39505669–SRR39505828.

## SUPPLEMENTAL MATERIAL

Supplemental material aem.01078-26-s0001.docx Graphical abstract, Tables S1 to S14, Text S1, and Fig. S1 to S9.

ASM does not own the copyrights to Supplemental Material that may be linked to, or accessed through, an article. The authors have granted ASM a non-exclusive, world-wide license to publish the Supplemental Material files. Please contact the corresponding author directly for reuse.
