# Supplementary material for: Disinfection and damage of nontuberculous mycobacteria by different UV wavelengths
Source: Appl Environ Microbiol. 2026 Jul 31;92(8):e01078-26. doi: 10.1128/aem.01078-26 (PMC13488321; doi:10.1128/aem.01078-26)
Supplement: Supplementary file 1 [file aem.01078-26-s0001.docx]

**Disinfection and damage of nontuberculous mycobacteria by different UV wavelengths**

Yijing Liu^1^, Eunice Kum^2^, Richard Robinson^3^, Natalie Hull^1,4^

^1^ College of Engineering, Department of Civil, Environmental and Geodetic Engineering, The Ohio State University, Columbus, OH 43210, USA

^2^ College of Arts and Sciences, Department of Microbiology, The Ohio State University, Columbus, OH 43210, USA

^3^ Department of Microbial Infection and Immunity, The Ohio State University, Columbus, OH 43210, USA

^4^The Sustainability Institute, The Ohio State University, Columbus, OH, 43210, USA

*Corresponding author: [hull.305@osu.edu](mailto:hull.305@osu.edu), current email: [nataliehull@boisestate.edu](mailto:nataliehull@boisestate.edu)

**Graphical Abstract:**

Visual overview of the study design and experimental workflow

Table S1 Summary of four parameter log logistic model estimates with their corresponding standard error depicting the growth curve of OD at 600 nm over time curve for each *M. smegmatis* strain and each morphotype of M. intracellulare.

| **NTM** | **b (1/hour)** | **d (OD_600_)** | **e (hour)** |
| --- | --- | --- | --- |
| M. intracellulare _Parent | -4.06±2.20 | 0.24±0.05 | 135.94±21.94 |
| M. intracellulare _Smooth | -3.87±1.35 | 0.36±0.07 | 158.00±21.46 |
| M. intracellulare _Rough | -6.38±2.61 | 0.24±0.06 | 167.37±15.84 |
| *M. smegmatis* 19420 | -4.27±1.50 | 0.54±0.05 | 26.34±2.02 |
| *M. smegmatis* 14468 | -8.44±1.86 | 0.39±0.016 | 28.85±0.83 |

Table S2 Summary of slope and intercept of linear model with their corresponding standard error of the estimate depicting the growth curve of Log_10_ CFU/mL over time for each *M. smegmatis* strain and each morphotype of M. intracellulare.

| **NTM** | **Slope (log_10_CFU/mL/hour)** | **Intercept (log_10_CFU/mL)** |
| --- | --- | --- |
| M. intracellulare _Parent | 0.02±0.001 | 5.04±0.24 |
| M. intracellulare _Smooth | 0.01±0.001 | 5.64±0.23 |
| M. intracellulare _Rough | 0.02±0.003 | 6.02±0.51 |
| *M.Smegmatis* 19420 | 0.05±0.004 | 5.91±0.16 |
| *M.Smegmatis* 14468 | 0.06±0.004 | 5.47±0.17 |

Table S3:  Collection time and OD value at 600nm (OD_600_) for each replicate of *M. Smegmatis* 19420. OD_600_ was averaged value for three technical replicates with their corresponding standard deviation.

| Replicate 1:  Date: Nov 11, 2022 | | Replicate 2:  Date: June 1, 2023 | | Replicate 3:  Date: June 6, 2023 | |
| --- | --- | --- | --- | --- | --- |
| Duration (hr) | OD_600_ | Duration (hr) | OD_600_ | Duration (hr) | OD_600_ |
| 0 | 0.007 ± 0 | 0 | 0.004 ± 0.001 | 0 | 0.007 ± 0.001 |
| 5.5 | 0.006 ± 0 | 4.02 | 0.003 ± 0.001 | 3.83 | 0.009 ± 0.002 |
| 8 | 0.011 ± 0.003 | 9.83 | 0.033 ± 0.020 | 9.83 | 0.014 ± 0.002 |
| 17.5 | 0.053 ± 0.004 | 22.06 | 0.129 ± 0.045 | 22.1 | 0.078 ± 0.002 |
| 20.5 | 0.133 ± 0.03 | 24.93 | 0.202 ± 0.047 | 24.92 | 0.131 ± 0.017 |
| 23.5 | 0.389 ± 0.001 | 27.75 | 0.265 ± 0.020 | 27.88 | 0.213 ± 0.034 |
| 25.5 | 0.498 ± 0.017 | 30.82 | 0.323 ± 0.007 | 30.9 | 0.214 ± 0.005 |
| 29.667 | 0.560 ± 0.002 | 35.22 | 0.378 ± 0.021 | 46.15 | 0.324 ± 0.015 |
| 32.75 | 0.311 ± 0.385 | 45.78 | 0.480 ± 0.010 | 48.83 | 0.363 ± 0.014 |
| 44 | 0.642 ± 0.011 | 48.82 | 0.548 ± 0.034 | 51.75 | 0.395 ± 0.077 |
| 46 | 0.638 ± 0.01 | 51.82 | 0.467 ± 0.020 | 55.17 | 0.456 ± 0.004 |
| 50.083 | 0.662 ± 0.001 | 54.57 | 0.541 ± 0.020 |  |  |
| 53.833 | 0.661 ± 0.013 | 59.54 | 0.526 ± 0.046 |  |  |
| 57.833 | 0.687 ± 0.004 | 84.87 | 0.496 ± 0.023 |  |  |

Table S4:  Collection time and OD value at 600nm for each replicate of *M. Smegmatis* 14468. OD_600_ was averaged value for three technical replicates with their corresponding standard deviation.

| Replicate 1:  Date: Nov 11, 2022 | | Replicate 2:  Date: June 1, 2023 | | Replicate 3:  Date: June 6, 2023 | |
| --- | --- | --- | --- | --- | --- |
| Duration (hr) | OD_600_ | Duration (hr) | OD_600_ | Duration (hr) | OD_600_ |
| 0 | 0.009 ± 0.001 | 0 | 0.006 ± 0.003 | 0 | 0.006 ± 0 |
| 5.5 | 0.008 ± 0.001 | 4.02 | 0.003 ± 0.002 | 3.83 | 0.007 ± 0.001 |
| 8 | 0.007 ± 0.002 | 9.83 | 0.009 ± 0.044 | 9.83 | 0.007 ± 0.001 |
| 17.5 | 0.014 ± 0.001 | 22.06 | 0.069 ± 0.050 | 22.1 | 0.055 ± 0.004 |
| 20.5 | 0.018 ± 0.001 | 24.93 | 0.132 ± 0.030 | 24.92 | 0.091 ± 0.007 |
| 23.5 | 0.029 ± 0.001 | 27.75 | 0.243 ± 0.026 | 27.88 | 0.176 ± 0.013 |
| 25.5 | 0.049 ± 0.004 | 30.82 | 0.280 ± 0.016 | 30.9 | 0.227 ± 0.047 |
| 29.667 | 0.130 ± 0.004 | 35.22 | 0.546 ± 0.338 | 46.15 | 0.292 ± 0.003 |
| 32.75 | 0.281 ± 0.021 | 45.78 | 0.339 ± 0.011 | 48.83 | 0.312 ± 0.026 |
| 44 | 0.535 ± 0.054 | 48.82 | 0.363 ± 0.076 | 51.75 | 0.362 ± 0.068 |
| 46 | 0.517 ± 0.023 | 51.82 | 0.330 ± 0.028 | 55.17 | 0.346 ± 0.025 |
| 50.083 | 0.495 ± 0.026 | 54.57 | 0.367 ± 0.040 |  |  |
| 53.833 | 0.454 ± 0.090 | 59.54 | 0.407 ± 0.058 |  |  |
| 57.833 | 0.385 ± 0.061 | 84.87 | 0.310 ± 0.073 |  |  |

Table S5: Collection time and OD value at 600nm for each replicate of M. intracellulare Parent morphotype. OD_600_ was averaged value for three technical replicates with their corresponding standard deviation.

| Replicate 1  Date: Nov 11, 2022 | | Replicate 2:  Date: March 22, 2023 | | Replicate 3:  Date: June 1, 2023 | | Replicate 4:  Date: Sep 26, 2023 | |
| --- | --- | --- | --- | --- | --- | --- | --- |
| Duration (hr) | OD_600_ | Duration (hr) | OD_600_ | Duration (hr) | OD_600_ | Duration (hr) | OD_600_ |
| 0 | 0.008 ± 0.002 | 0 | 0.007 ±  0 | 0 | 0.006 ± 0.001 | 0 | 0.008 ± 0.001 |
| 25 | 0.009 ± 0.001 | 23.67 | 0.010 ± 0.003 | 24.93 | 0.009 ± 0.001 | 24.32 | 0.008 ± 0.001 |
| 73 | 0.091 ± 0.003 | 48 | 0.011 ± 0.002 | 48.82 | 0.008 ± 0.001 | 76.98 | 0.045 ± 0.003 |
| 101.25 | 0.271 ± 0.119 | 70.67 | 0.038 ± 0.002 | 84.87 | 0.007 ± 0.002 | 104.83 | 0.061 ± 0.005 |
| 129 | 0.198 ± 0.028 | 94.17 | 0.138 ± 0.002 | 96.82 | 0.009 ± 0 | 123.55 | 0.078 ± 0.005 |
| 153 | 0.203 ± 0.011 | 119.67 | 0.147 ± 0.005 | 102.92 | 0.010 ± 0.002 | 146.83 | 0.127 ± 0.006 |
| 170.5 | 0.220 ± 0.001 | 144.17 | 0.126 ± 0.002 | 120.87 | 0.014 ± 0.001 | 200.62 | 0.153 ± 0.013 |
| 200.5 | 0.345 ± 0.031 | 150.17 | 0.244 ± 0.009 | 126.75 | 0.014 ± 0.004 | 225.92 | 0.214 ± 0.011 |
| 217.5 | 0.367 ± 0.033 | 166.67 | 0.294 ± 0.008 | 144.93 | 0.016 ± 0.004 | 248.42 | 0.210 ± 0.023 |
|  |  | 173.42 | 0.258 ± 0.004 | 150.92 | 0.017 ± 0.006 | 269.72 | 0.211 ± 0.025 |
|  |  | 191.17 | 0.310 ± 0.002 | 168.85 | 0.033 ± 0.006 |  |  |
|  |  | 198.17 | 0.447 ± 0.025 | 175.18 | 0.041 ± 0.003 |  |  |
|  |  | 214.92 | 0.496 ± 0.026 | 216.98 | 0.080 ± 0.010 |  |  |
|  |  | 221.17 | 0.368 ± 0.005 | 222.93 | 0.103 ± 0.022 |  |  |
|  |  | 237.92 | 0.428 ± 0.012 | 240.78 | 0.189 ± 0.010 |  |  |
|  |  |  |  | 246.80 | 0.158 ± 0.010 |  |  |

Table S6: Collection time and OD value at 600nm for each replicate of M. intracellulare Smooth morphotype. OD_600_ was averaged value for three technical replicates with their corresponding standard deviation.

| Replicate 1  Date: Nov 11, 2022 | | Replicate 2:  Date: March 22, 2023 | | Replicate 3:  Date: June 1, 2023 | | Replicate 4:  Date: Sep 26, 2023 | |
| --- | --- | --- | --- | --- | --- | --- | --- |
| Duration (hr) | OD_600_ | Duration (hr) | OD_600_ | Duration (hr) | OD_600_ | Duration (hr) | OD_600_ |
| 0 | 0.006 ± 0.001 | 0 | 0.007 ± 0 | 0 | 0.004 ± 0.001 | 0 | 0.008 ± 0.002 |
| 25 | 0.007 ± 0.001 | 23.67 | 0.006 ± 0.005 | 24.93 | 0.007 ± 0.001 | 24.32 | 0.008 ± 0.003 |
| 73 | 0.113 ± 0.014 | 48 | 0.008 ± 0.003 | 48.82 | 0.008 ± 0.001 | 76.98 | 0.038 ± 0.007 |
| 101.25 | 0.151 ± 0.025 | 70.67 | 0.026 ± 0.004 | 84.87 | 0.006 ± 0.001 | 104.83 | 0.070 ± 0.007 |
| 129 | 0.351 ± 0.050 | 94.17 | 0.114 ± 0.004 | 96.82 | 0.010 ± 0.001 | 123.55 | 0.082 ± 0.005 |
| 153 | 0.250 ± 0.025 | 119.67 | 0.167 ± 0.011 | 102.92 | 0.015 ± 0.002 | 146.83 | 0.126 ± 0.009 |
| 170.5 | 0.272 ± 0.007 | 144.17 | 0.153 ± 0.005 | 120.87 | 0.028 ± 0.003 | 200.62 | 0.189 ± 0.004 |
| 200.5 | 0.368 ± 0.006 | 150.17 | 0.228 ± 0.020 | 126.75 | 0.036 ± 0.007 | 225.92 | 0.224 ± 0.019 |
| 217.5 | 0.320 ± 0.002 | 166.67 | 0.152 ± 0.019 | 144.93 | 0.086 ± 0.010 | 248.42 | 0.248 ± 0.030 |
|  |  | 173.42 | 0.299 ± 0.003 | 150.92 | 0.098 ± 0.003 | 269.72 | 0.233 ± 0.009 |
|  |  | 191.17 | 0.376 ± 0.003 | 168.85 | 0.106 ± 0.016 |  |  |
|  |  | 198.17 | 0.333 ± 0.019 | 175.18 | 0.192 ± 0.003 |  |  |
|  |  | 214.92 | 0.385 ± 0.052 | 216.98 | 0.188 ± 0.023 |  |  |
|  |  | 221.17 | 0.349 ± 0.008 | 222.93 | 0.267 ± 0.006 |  |  |
|  |  | 237.92 | 0.452 ± 0.014 | 240.78 | 0.237 ± 0.027 |  |  |
|  |  |  |  | 246.8 | 0.264 ± 0.033 |  |  |
|  |  |  |  | 264.9 | 0.360 ± 0.012 |  |  |
|  |  |  |  | 271.65 | 0.367 ± 0.029 |  |  |
|  |  |  |  | 288.93 | 0.304 ± 0.017 |  |  |

Table S7: Collection time and OD value at 600nm for each replicate of M. intracellulare Rough morphotype. OD_600_ was averaged value for three technical replicates with their corresponding standard deviation.

| Replicate 1  Date: Nov 11, 2022 | | Replicate 2:  Date: March 22, 2023 | | Replicate 4:  Date: Sep 26, 2023 | |
| --- | --- | --- | --- | --- | --- |
| Duration (hr) | OD_600_ | Duration  (hr) | OD_600_ | Duration (hr) | OD_600_ |
| 0 | 0.006 ± 0.003 | 0 | 0.006 ± 0 | 0 | 0.007 ± 0 |
| 25 | 0.007 ± 0.001 | 23.67 | 0.007 ± 0.005 | 24.93 | 0.006 ± 0.002 |
| 73 | 0.113 ± 0.001 | 48 | 0.006 ± 0.004 | 50.38 | 0.008 ± 0.001 |
| 101.25 | 0.151 ± 0.006 | 70.67 | 0.009 ± 0.005 | 67.78 | 0.030 ± 0.007 |
| 129 | 0.351 ± 0.002 | 94.17 | 0.016 ± 0.003 | 94.83 | 0.031 ± 0.006 |
| 153 | 0.250 ± 0.006 | 119.67 | 0.016 ± 0.006 | 124.97 | 0.046 ± 0.005 |
| 170.5 | 0.272 ± 0.011 | 144.17 | 0.025 ± 0.005 | 188.8 | 0.095 ± 0.005 |
| 200.5 | 0.368 ± 0.004 | 150.17 | 0.035 ± 0.005 | 212.92 | 0.111 ± 0.013 |
| 217.5 | 0.320 ± 0.008 | 166.67 | 0.043 ± 0.006 | 237.08 | 0.237 ± 0.019 |
|  |  | 173.42 | 0.092 ± 0.008 | 261.33 | 0.212 ± 0.017 |
|  |  | 191.17 | 0.138 ± 0.004 |  |  |
|  |  | 198.17 | 0.136 ± 0.053 |  |  |
|  |  | 214.92 | 0.207 ± 0.035 |  |  |
|  |  | 221.17 | 0.139 ± 0.010 |  |  |
|  |  | 237.92 | 0.276 ± 0.027 |  |  |

Table S8:  Collection time and CFU value for each replicate of *M. Smegmatis* 19420 and 14468. Averaged value for 10 technical replicates with their corresponding standard deviation was shown.

| Replicate 1:  Date: Nov 11, 2022 | | | Replicate 2:  Date: June 1, 2023 | | | Replicate 3:  Date: June 6, 2023 | | |
| --- | --- | --- | --- | --- | --- | --- | --- | --- |
| Duration (hr) | 19420 CFU | 14468 CFU | Duration (hr) | 19420 CFU | 14468 CFU | Duration (hr) | 19420 CFU | 14468 CFU |
| 0 | 2.07E+05 | 4.60E+04 | 0 | 2.44E+05  (± 6.29) | 1.11E+05  (± 4.15) | 0 | 1.74E+05  (± 5.17) | 7.20E+04  (± 2.86) |
| 5.5 | 4.40E+05 | 5.20E+05 | 4.02 | 2.60E+05  (± 5.48) | 2.32E+05  (± 5.35) | 3.83 | 2.36E+05  (± 4.25) | 3.17E+05  (± 6.91) |
| 8 | 3.68E+06 | 1.39E+06 | 9.83 | 1.38E+06  (± 4.08) | 1.62E+06  (± 2.86) | 9.83 | 8.70E+05  (± 3.80) | 1.06E+06  (± 3.72) |
| 17.5 | 2.49E+07 | 6.20E+06 | 22.06 | 1.13E+07  (± 3.86) | 5.50E+06  (± 1.72) | 22.1 | 1.18E+07  (± 3.01) | 8.50E+06  (± 3.06) |
| 20.5 | 2.72E+07 | 9.80E+06 | 24.93 | 4.90E+07  (± 2.23) | 1.60E+07  (± 1.43) | 24.92 | 1.88E+07  (± 4.34) | 1.23E+07  (± 4.92) |
| 23.5 | 2.52E+07 | 1.20E+07 | 27.75 | 1.17E+08  (± 3.13) | 4.00E+07  (± 2.79) | 27.88 | 7.10E+07  (± 2.28) | 1.92E+07  (± 4.80) |
| 25.5 | 3.80E+07 | 2.02E+07 | 30.82 | 1.75E+08  (± 8.10) | 1.19E+08  (± 2.64) | 30.9 | 1.37E+08  (± 2.98) | 1.59E+08  (± 3.75) |
| 29.67 | 5.80E+07 | 3.20E+06 | 35.22 | 1.49E+08  (± 3.84) |  | 46.15 |  | 1.22E+09  (± 6.61) |
| 32.75 | 2.37E+08 | 5.30E+06 | 45.78 | 3.36E+08  (± 3.78) | 8.70E+08  (± 3.80) | 48.83 | 2.91E+08  (± 5.65) | 9.10E+08  (± 2.73) |
| 44 | 3.10E+08 | 8.60E+08 | 48.82 | 2.89E+08  (± 5.07) | 9.90E+08  (± 3.35) | 51.75 | 3.41E+08  (± 1.84) | 3.90E+08  (± 2.38) |
| 46 | 2.90E+08 | 1.88E+09 | 51.82 | 3.56E+08  (± 4.35) | 8.20E+08  (± 2.39) | 55.17 | 1.16E+09  (± 7.18) | 1.07E+09  (± 4.66) |
| 50.08 | 1.90E+08 | 1.70E+09 | 54.57 | 3.51E+08  (± 8.56) | 7.60E+08  (± 2.91) |  |  |  |
| 53.83 | 6.00E+08 | 1.27E+09 | 59.54 | 3.85E+08  (± 4.86) | 6.00E+08  (± 2.21) |  |  |  |
|  |  |  | 84.87 | 4.80E+08  (± 2.04) | 7.00E+08  (± 2.26) |  |  |  |

Table S9:  Collection time and CFU value for each replicate of M. intracellulare parent, smooth and rough morphotype. Averaged value for 10 technical replicates with their corresponding standard deviation was shown.

| Replicate 1  Date: Nov 11, 2022 | | | | Replicate 2:  Date: March 22, 2023 | | | |
| --- | --- | --- | --- | --- | --- | --- | --- |
| Duration (hr) | Smooth CFU | Rough CFU | Parent CFU | Duration (hr) | Smooth CFU | Rough CFU | Parent CFU |
| 0 | 5.80E  +05 | 3.00E  +05 | 2.60E  +04 | 0 | 5.80E+05  (± 0) | 5.43E+04  (± 0) | 2.60E+04  (± 0) |
| 25 | 1.50E  +06 | 6.90E  +05 | 9.40E  +05 | 23.67 | 4.24E+05  (± 5.27) | 4.89E+04  (± 12.2) | 3.08E+05  (± 4.55) |
| 73 | 2.64E  +07 | 2.64E  +07 | 2.40E  +06 | 48 | 5.52E+05  (± 4.64) | 7.40E+04  (± 2.41) | 5.94E+05  (± 5.70) |
| 101.25 | 3.28E  +08 | 2.16E  +09 | 3.70E  +06 | 70.67 | 1.38E+06  (± 5.16) | 1.20E+05  (± 6.27) | 1.94E+06  (± 4.20) |
| 129 | 3.70E  +07 | 1.00E  +09 | 2.20E  +07 | 94.17 | 5.39E+06  (± 8.82) | 4.14E+05  (± 6.64) | 7.40E+06  (± 10.3) |
| 153 | 1.31E  +09 | 2.00E  +09 | 2.62E  +08 | 119.67 | 3.28E+08  (± 6.61) | 8.70E+05  (± 2.54) | 1.55E+08  (± 3.41) |
| 170.5 | 2.70E  +09 | 1.94E  +09 | 9.00E  +08 | 144.17 | 2.97E+08  (± 4.64) | 2.70E+06  (± 0) | 7.20E+08  (± 2.66) |
| 200.5 | 2.15E  +09 | 2.60E  +09 | 7.90E  +08 | 150.17 | 2.13E+08  (± 4.90) | 3.48E+06  (± 10.15) | 3.02E+08  (± 3.33) |
| 217.5 | 3.40E  +09 |  | 6.40E  +08 | 166.67 | 4.50E+08  (± 1.35) | 5.50E+06  (± 0) | 9.10E+08  (± 3.11) |
|  |  |  |  | 173.42 | 4.07E+08  (± 9.86) | 6.10E+06  (± 1.45) | 4.53E+08  (± 5.91) |
|  |  |  |  | 191.17 | 6.20E+08  (± 2.66) | 1.73E+07  (± 4.19) | 1.14E+09  (± 3.37) |
|  |  |  |  | 198.17 | 7.50E+08  (± 3.69) | 2.10E+07  (± 0) | 1.60E+09  (± 3.16) |
|  |  |  |  | 214.92 | 3.39E+08  (± 3.07) | 4.77E+07  (± 3.31) |  |
|  |  |  |  | 221.17 | 6.80E+08  (± 0) | 6.63E+07  (± 10.47) | 7.00E+08  (± 0) |
|  |  |  |  | 237.92 | 7.50E+08  (± 0) | 2.42E+08  (± 4.85) | 1.75E+09  (± 2.81) |

| Replicate 3:  Date: June 1, 2023 | | | Replicate 4:  Date: Sep 26, 2023 | | | |
| --- | --- | --- | --- | --- | --- | --- |
| Duration (hr) | Smooth CFU | Parent CFU | Duration (hr) | Smooth CFU | Rough CFU | Parent CFU |
| 0 | 2.81E+05  (± 4.82) | 2.46E+05  (± 3.66) | 0 | 5.02E+05  (± 13.6) | 4.10E+04  (± 2.13) | 9.40E+05  (± 2.80) |
| 24.93 | 2.85E+05  (± 3.31) | 2.63E+05  (± 4.16) | 24.32 | 6.10E+05  (± 3.07) | 1.77E+05  (± 4.55) | 5.10E+05  (± 2.60) |
| 48.82 | 3.43E+07  (± 4.85) | 3.68E+05  (± 5.89) | 76.98 | 3.66E+06  (± 7.62) | 4.00E+05  (± 1.56) | 1.76E+07  (± 4.25) |
| 84.87 | 3.40E+05  (± 1.84) | 2.90E+05  (± 1.91) | 104.83 | 4.39E+07  (± 11.37) | 2.66E+06  (± 6.28) |  |
| 96.82 | 1.33E+06  (± 3.02) | 5.70E+05  (± 2.21) | 123.55 |  | 6.30E+06  (± 2.63) |  |
| 102.92 | 2.04E+06  (± 5.78) | 7.10E+05  (± 1.91) | 146.83 |  |  |  |
| 120.87 | 5.14E+06  (± 7.44) | 1.74E+06  (± 2.21) | 200.62 | 1.25E+08  (± 4.25) |  | 1.15E+08  (± 1.72) |
| 126.75 | 4.30E+06  (± 2.21) | 2.04E+06  (± 1.91) | 225.92 | 1.84E+08  (± 4.72) |  | 1.37E+08  (± 4.76) |
| 144.93 | 1.21E+07  (± 3.14) | 1.60E+06  (± 5.13) | 248.42 | 2.11E+08  (± 3.67) |  | 1.08E+08  (± 6.22) |
| 150.92 | 2.08E+07  (± 6.29) | 3.40E+06  (± 8.17) | 269.72 | 2.48E+08  (± 8.92) |  | 2.27E+08  (± 3.59) |

Table S10: Forward and reverse primer sequence for specific genes

| Gene | Primer (5’ – 3’) | Fragment size (bp) | References |
| --- | --- | --- | --- |
| *rpoB*  *hsp65* | F: GGCAAGGTCACCCCGAAGGG  R:AGCGGCTGCTGGGTGATCATC  F: ACCAACGATGGTGTGTCCAT  R: CTTGTCGAACCGCATACCCT | 723  441 | (Meghdadi et al., 2022)  (Van Der Wielen et al., 2013) |

Table S11: Cycling conditions for *rpoB*

| Stage | Time | Temperature | Cycle number |
| --- | --- | --- | --- |
| Initial denaturation | 4 mins | 95 °C | 1 |
| Denaturation | 45 secs | 95 °C | 35 |
| Annealing | 20 secs | 58 °C | 35 |
| Extension | 20 secs | 72 °C | 35 |
| Final extension | 10 mins | 72 °C | 1 |
| Hold |  | 4 °C |  |

Table S12: Cycling conditions for *hsp65*

| Stage | Time | Temperature | Cycle number |
| --- | --- | --- | --- |
| Initial denaturation | 2 mins | 95 °C | 1 |
| Denaturation | 30 secs | 95 °C | 43 |
| Annealing | 1 min | 60 °C | 43 |
| Extension | 1 min | 72 °C | 43 |
| Final extension | 7 mins | 72 °C | 1 |
| Hold |  | 4 °C |  |

Text S1: Sequencing information of designed gBlocks for *rpoB* and *hsp65.* Colours indicate primer binding sites.

***rpoB* gBlock design:**

GACTGATGCTCAGTGAGTTACTACGCAGTCACTCAGTCGGCAAGGTCACCCCGAAGGGTGAGACCGAGCTGACCCCGGAGGAGCGGCTGCTCCGCGCGATCTTCGGTGAGAAGGCGCGCGAGGTCCGCGACACGTCGCTGAAGGTCCCGCACGGTGGAACCGTTCCCGTACCCGGTGACGGTTGGCTACATGTACATCCTGAAGCTGCACCACCTGGTCGACGACAAGATCCACGCGCGTTCGACCGGTCCGTACTCGATGATCACCCAGCAGCCGCTCGGACACTTAGACCAGATGTCCGTGACGTCTAGCTTGA

***hsp65*** **gBlock design:**

GACTGATGCTCAGTGAGTTACTACGCAGTCACTCACCCCACGATCACCAACGATGGTGTGTCCATCGCCAAGGAGATCGAGCTGGAGGACCCGTACGAGAAGATCGGCGCCGAGCTGGTCAAGGAAGTCGCCAAGAAGATGGGTCTCAAGCGCGGCATCGAGAAGGCCGTCGAGAAGGTCACCGAGACCCTGCTCAAGTCGGCCAAGGAGGTCGAGACCAAGGACCAGATCGCTGCCACCGCGGCGATTTCGGCGGGCGACCAGTCGATCGGTGACCTCATCGCCGAGGCGATGGACAAGGTCGGCAACGAGGGCGTCATCACCGTCGAGGAGTCCAACACCTTCGGCCTGCAGCTCGAGCTCACCGAGGGTATGCGGTTCGACAAGGGCACACTTAGACCAGATGTCCGTGACGTCTAGCTTGA

Table S13 Summary of three parameter with their corresponding standard deviation of log logistic model applied to log10 inactivation in UV dose curve for each *M. smegmatis* strain and each morphotype of M. intracellulare under different UV wavelengths.

| NTM | UV wavelength | b (cm^2^/mJ per log inactivation) | d (log reduction) | e (mJ/cm^2^) | D_1_ (mJ/cm^2^) | D_2_ (mJ/cm^2^) | k_1_ (cm^2^/mJ) | k_2_  (cm^2^/mJ) | EEO@ D_1_ (kwh/m^3^) |
| --- | --- | --- | --- | --- | --- | --- | --- | --- | --- |
| *M. smegmatis* 19420 | 254 nm | -4.11±1.96 | 4.04±0.27 | 12.11±1.24 | 9.24 | 12.05 | 0.1082 | 0.0830 | 3.08E-03 |
|  | 222 nm | -6.96±3.20 | 3.03±0.16 | 12.47±0.71 | 11.26 | 13.72 | 0.0888 | 0.0729 | 1.36E-02 |
| *M. smegmatis* 14468 | 254 nm | -4.08±1.23 | 3.57±0.14 | 12.45±0.71 | 9.88 | 13.21 | 0.1012 | 0.0757 | 3.04E-03 |
|  | 222 nm | -2.98±0.88 | 3.33±0.13 | 13.37±0.98 | 10.07 | 15.33 | 0.0993 | 0.0652 | 3.28E-03 |
| M. intracellulare _Parent | 254 nm | -7.49±1.03 | 2.88±0.10 | 13.84±0.75 | 12.72 | 15.44 | 0.0786 | 0.0648 | 3.59E-03 |
|  | 222 nm | -4.38±2.76 | 2.72±0.15 | 13.58±0.76 | 12.00 | 17.15 | 0.0833 | 0.0583 | 3.61E-03 |
| M. intracellulare _Smooth | 254 nm | -2.63±0.90 | 3.67±0.20 | 14.40±1.52 | 9.91 | 15.42 | 0.1009 | 0.0648 | 1.02E-02 |
|  | 222 nm | -4.10±0.83 | 2.99±0.08 | 13.44±0.59 | 11.36 | 15.95 | 0.0880 | 0.0627 | 1.24E-02 |
| M. intracellulare _Rough | 254 nm | -5.16±1.44 | 2.61±0.10 | 14.14±0.73 | 12.89 | 17.8 | 0.0776 | 0.0562 | 1.16E-02 |
|  | 222 nm | -1.02±0.72 | 3.86±2.36 | 34.40±50.18 | 12.28 | 36.94 | 0.0814 | 0.0271 | 1.12E-02 |

Table S14 Summary of three parameter with their corresponding standard deviation of log logistic model applied to log10 gene damage of *rpoB* and *hsp65* in UV dose curve with each *M. smegmatis* strain and each morphotype of M. intracellulare under different UV wavelengths.

| Strain | UV wavelength | Gene | b (cm^2^/mJ per log gene damage) | d (log reduction) | e (mJ/cm^2^) | D_1_ (mJ/cm^2^) | k_1_ (cm^2^/mJ) | EEO@ D_1_ (kwh/m^3^) |
| --- | --- | --- | --- | --- | --- | --- | --- | --- |
| *M. smegmatis* 19420 | 254 nm | *rpoB* | -1.08±0.48 | 2.46±2.81 | 116.55±230.32 | 82.10 | 0.012 | 2.74E-02 |
|  | 222 nm |  | -3.49±3.05 | 1.08±0.12 | 17.86±3.99 | 36.83 | 0.027 | 1.28E-02 |
| *M. smegmatis* 14468 | 254 nm |  | -1.27±0.69 | 1.38±0.50 | 30.41±23.38 | 65.15 | 0.015 | 2.00E-02 |
|  | 222 nm |  | -2.13±0.62 | 1.56±0.14 | 19.13±4.45 | 25.12 | 0.040 | 8.19E-03 |
| M. intracellulare _Parent | 254 nm |  | 39.74±37.73 | 0.80±0.09 | 69.96±9.60 | NA | NA | NA |
|  | 222 nm |  | -0.58±0.15 | 16.16±38.28 | 2396.83±12886.20 | 22.08 | 0.045 | 2.32E-02 |
| M. intracellulare _Smooth | 254 nm |  | -0.77±0.39 | 4.09±15.64 | 901.38±5852.69 | 208.26 | 0.005 | 2.14E-01 |
|  | 222 nm |  | -0.76±0.3s7 | 6.21±20.61 | 870.76±5334.31 | 99.24 | 0.010 | 1.08E-01 |
| M. intracellulare _Rough | 254 nm |  | -1.37±0.41 | 1.43±0.39 | 42.83±22.98 | 79.30 | 0.013 | 2.03E-02 |
|  | 222 nm |  | -3.28±3.06 | 1.06±0.26 | 26.64±15.40 | 62.81 | 0.016 | 5.75E-02 |
| *M. smegmatis* 19420 | 254 nm | *hsp65* | -0.62±0.29 | 3.2±8.42 | 627.92±3932.90 | 176.04 | 0.006 | 5.86E-02 |
|  | 222 nm |  | -0.81±0.3 | 4.28±6.7 | 305.46±955.57 | 70.48 | 0.014 | 8.54E-02 |
| *M. smegmatis* 14468 | 254 nm |  | -2.18±1.41 | 1.07±0.22 | 18.75±9.38 | 63.50 | 0.016 | 1.95E-02 |
|  | 222 nm |  | -0.67±0.09 | 13.98±13.89 | 2495.35±4418.00 | 54.39 | 0.018 | 6.21E-02 |
| M. intracellulare _Parent | 254 nm |  | -1.21±0.37 | 1.42±0.53 | 53.02±37.99 | 108.59 | 108.59 | 3.07E-02 |
|  | 222 nm |  | -0.82±0.28 | 18.64±40.68 | 2387.50±6843.56 | 72.08 | 72.08 | 7.59E-02 |
| M. intracellulare _Smooth | 254 nm |  | -1.44±0.76 | 1.19±0.36 | 28.29±17.30 | 89.64 | 89.64 | 2.63E-02 |
|  | 222 nm |  | -0.59±0.31 | 3.61±6.97 | 401.66±2132.41 | 79.01 | 79.01 | 8.63E-02 |
| M. intracellulare _Rough | 254 nm |  | -0.46±0.45 | 5.28±13.75 | 3274.51±17182.29 | 138.82 | 138.82 | 3.55E-02 |
|  | 222 nm |  | -0.67±0.09 | 11.64±14.86 | 1602.25±3971.21 | 46.99 | 46.99 | 4.30E-02 |

M.Smegmatis_14468

M.Smegmatis_19420

M. intracellulare_Parent

M. intracellulare_Rough

M. intracellulare_Smooth

Figure S1 Growth curves (OD vs. time) for *M. smegmatis* (green) and different morphotypes of M. intracellulare (red) with three biological replicates. Error bars represent the standard deviations of three technical replicates of each strain. 5 missing error bars with wide range cannot show in the figure. Solid red line represents M. intracellulare _Parent, dashed red line represents MAC_Rough while dotted red line represents M. intracellulare_Smooth. Solid green line represents *M. smegmatis* 19420 while dashed green line represents *M. smegmatis* 14468.

M.Smegmatis_14468

M.Smegmatis_19420

M. intracellulare_Parent

M. intracellulare_Rough

M. intracellulare_Smooth

Figure S2 Growth curves (Log_10_ CFU/mL vs. time) for *M. smegmatis* (green) and different morphotypes of M. intracellulare (red)*.* Growth curves were modeled with linear model fitted to the log transformed colony forming units (CFU) data in R Studio. Error bars represent the standard deviations of 10 technical replicates of each strain. Solid red line represents M. intracellulare _Parent, dashed red line represents M. intracellulare _Rough while dotted red line represents M. intracellulare _Smooth. Solid green line represents *M. smegmatis* 19420 while dashed green line represents *M. smegmatis* 14468.

**Figure S3** Standard curves for *rpoB* gene as a function of gblock concentration dilution factor and extracted NTM stock dilution factor. Error bars represent standard deviations of three technical replicates at each dilution factor. Dilution factor 0 represents the undiluted copy numbers of *rpoB* gblock (3.09*10^10 copies/μl). The undiluted copy number for *rpoB* was 3.09*10^10^ copies/ml, calculated by C*M*(1*10^-15^ mol/fmol)* N_A_,where C is the concentration of the gene fragment (10 ng/μl) , M is the molecular weight provided by IDT (5.12 fmoles/ng), and N_A_ = 6.022* 10^23^ mol^-1^.

**Figure S4** Standard curves for *hsp65* gene as a function of gblock concentration dilution factor and extracted NTM stock dilution factor. Error bars represent standard deviations of three technical replicates at each dilution factor. Dilution factor 0 represents the undiluted copy numbers of *rpoB* gblock (2.3*10^10 copies/μl). The undiluted copy number for *hsp65* was 2.30*10^10^ copies/ml, calculated by C*M*(1*10^-15^ mol/fmol)* N_A_,where C is the concentration of the gene fragment (10 ng/μl) , M is the molecular weight provided by IDT (3.81 fmoles/ng), and N_A_ = 6.022* 10^23^ mol^-1^.

| (a) |  |  |  |  |  |  |
| --- | --- | --- | --- | --- | --- | --- |
|  | | | | | | |
| (b) | | | | | | |
|  | | | | | | |

**Figure S5 (a).** *rpoB gene* damage (222 nm, red vs. 254 nm, blue) of *M. Smegmatis* 19420 and 14468. **(b).** *hsp65* gene damage (222 nm, red vs. 254 nm, blue) of *M. Smegmatis* 19420 and 14468. Error bars represent SEM of three biological replicates at each UV dose.

***M. intracellulare_*Smooth**

***M. intracellulare_*Smooth**

***M. intracellulare_*Parent**

***M. intracellulare_*Rough**

| (a) |  |  |  |  |  |  |
| --- | --- | --- | --- | --- | --- | --- |
|  | | | | | | |
| (b)  ***M. intracellulare_*Rough**  ***M. intracellulare_*Parent** | | | | | | |
|  | | | | | | |

**Figure S6 (a).** *rpoB gene* damage (222 nm, red vs. 254 nm, blue) of M. intracellulare _Parent (left), M. intracellulare_Rough (middle) and M. intracellulare_Smooth (right). **(b).** *hsp65* gene damage (222 nm, red vs. 254 nm, blue) of M. intracellulare_Parent (left), M. intracellulare_Rough (middle) and M. intracellulare_Smooth (right). Error bars represent SEM of three biological replicates at each UV dose.

DNA Concentration (ng/ul)

| UV dose (mJ/cm2) | M. smegmatis 254nm | Parent 254 nm | Smooth 254 nm | Rough 254 nm | M. smegmatis 222 nm | Parent 222 nm | Smooth 222 nm | Rough 222 nm |
| --- | --- | --- | --- | --- | --- | --- | --- | --- |
| 0 | 12.95 | 13.02 | 17.96 | 13.25 | 10.15 | 3.01 | 12.93 | 13.25 |
| 2 | 8.3 | 8.26 | 8.32 | 9.1 | 3.86 | 8.32 | 8.28 | 9.1 |
| 4 | 5.4 | 5.35 | 5.46 | 6.78 | 3.62 | 5.43 | 5.42 | 6.78 |
| 8 | 2.59 | 2.63 | 2.54 | 3.42 | 2.23 | 2.56 | 2.56 | 1.42 |
| 12 | 4.5 | 4.45 | 4.56 | 5.67 | 6.65 | 4.48 | 4.53 | 5.67 |
| 16 | 2.5 | 2.47 | 2.53 | 3.25 | 5.55 | 2.52 | 2.49 | 3.25 |
| 40 | 2.16 | 2.13 | 2.14 | 2.89 | 3.07 | 2.17 | 3.18 | 2.89 |
| 60 | 1.21 | 1.26 | 1.2 | 1.77 | 2.25 | 1.22 | 1.25 | 2.77 |
| 80 | 1.13 | 1.19 | 1.16 | 1.32 | 1.85 | 1.15 | 2.11 | 2.32 |
| 120 | 1.05 | 1.09 | 3.04 | 2.09 | 2.02 | 1.07 | 1.07 | 1.09 |

**Figure S7** Comparison of DNA concentrations in *M. smegmatis* 19420, M. intracellulare parent, smooth and rough morphology before and after exposure to varying UV wavelengths and doses.

DNA Size (nucleotides)

| UV dose (mJ/cm2) | M. smegmatis 254 nm before | M. smegmatis 254 nm after | Parent 254 nm before | Parent 254 nm after | Smooth 254 nm before | Smooth 254 nm after | Rough 254 nm before | Rough 254 nm after |
| --- | --- | --- | --- | --- | --- | --- | --- | --- |
| 0 | 508 | 487 | 533 | 539 | 478 | 474 | 433 | 523 |
| 2 | 452 | 423 | 449 | 481 | 455 | 443 | 564 | 470 |
| 4 | 412 | 415 | 479 | 473 | 475 | 470 | 422 | 419 |
| 8 | 424 | 456 | 508 | 507 | 416 | 398 | 495 | 504 |
| 12 | 489 | 498 | 512 | 419 | 515 | 527 | 525 | 518 |
| 16 | 446 | 474 | 423 | 426 | 539 | 486 | 448 | 513 |
| 40 | 431 | 449 | 446 | 432 | 443 | 440 | 419 | 471 |
| 60 | 453 | 432 | 539 | 520 | 511 | 514 | 420 | 430 |
| 80 | 476 | 467 | 412 | 449 | 434 | 452 | 472 | 499 |
| 120 | 475 | 0 | 527 | 503 | 518 | 525 | 524 | 448 |

**Figure S8** Comparison of DNA size in *M. smegmatis* 19420, M. intracellulare parent, smooth and rough morphology after *rpoB* PCR but before and after library preparation with varying UV wavelengths and doses (254 nm).

DNA Size (nucleotides)

| UV dose (mJ/cm2) | M. smegmatis 222 nm before | M. smegmatis 222 nm after | Parent 222 nm before | Parent 222 nm after | Smooth 222 nm before | Smooth 222 nm after | Rough 222 nm before | Rough 222 nm after |
| --- | --- | --- | --- | --- | --- | --- | --- | --- |
| 0 | 539 | 481 | 443 | 412 | 426 | 457 | 532 | 545 |
| 2 | 482 | 415 | 547 | 466 | 568 | 521 | 498 | 480 |
| 4 | 458 | 402 | 565 | 504 | 548 | 465 | 479 | 531 |
| 8 | 464 | 458 | 422 | 437 | 532 | 415 | 451 | 458 |
| 12 | 452 | 445 | 559 | 519 | 540 | 512 | 524 | 509 |
| 16 | 451 | 448 | 521 | 416 | 510 | 544 | 529 | 518 |
| 40 | 457 | 459 | 528 | 477 | 525 | 476 | 539 | 523 |
| 60 | 450 | 447 | 465 | 453 | 471 | 535 | 413 | 419 |
| 80 | 455 | 433 | 570 | 537 | 504 | 408 | 434 | 465 |
| 120 | 444 | 414 | 506 | 416 | 543 | 476 | 505 | 416 |

**Figure S9** Comparison of DNA size in *M. smegmatis* 19420, M. intracellulare parent, smooth and rough morphology after *rpoB* PCR but before and after library preparation with varying UV wavelengths and doses (222 nm).
